# Impact of the dead-time correction method on quantitative ^177^Lu-SPECT (QSPECT) and dosimetry during radiopharmaceutical therapy

**DOI:** 10.1186/s40658-022-00484-w

**Published:** 2022-08-17

**Authors:** Alessandro Desy, Guillaume F. Bouvet, Nancy Lafrenière, Atefeh Zamanian, Philippe Després, Jean-Mathieu Beauregard

**Affiliations:** 1 Department of Medical Imaging, and Research Centre (Oncology Axis), CHU de Québec – Université Laval, 11 Côte du Palais, QC G1R 2J6 Quebec City, Canada; 2 Department of Radiology and Nuclear Medicine, and Cancer Research Centre, Université Laval, Quebec City, Canada; 3 Department of Radiation Oncology, and Research Centre (Oncology Axis), CHU de Québec – Université Laval, Quebec City, Canada; 4 Department of Physics, Physical Engineering and Optics, and Cancer Research Centre, Université Laval, Quebec City, Canada

**Keywords:** Quantitative imaging, Single-photon emission computed tomography, Dead time, Dosimetry, Lutetium-177, Radiopharmaceutical therapy

## Abstract

**Background:**

Dead-time correction is required for accurate quantitative SPECT-based dosimetry in the context of personalised ^177^Lu radiopharmaceutical therapy. We aimed to evaluate the impact of applying dead-time correction on the reconstructed SPECT image versus on the acquisition projections before reconstruction.

**Methods:**

Data from 16 SPECT/CT acquisitions of a decaying ^177^Lu-filled phantom (up to 20.75 GBq) and dual-timepoint SPECT/CT in 14 patients treated with personalised ^177^Lu peptide receptor radionuclide therapy were analysed. Dead time was determined based on the acquisition wide-spectrum count rate for each projection and averaged for the entire acquisition. Three dead-time correction methods (DTCMs) were used: the per-projection correction, where each projection was individually corrected before reconstruction (DTCM1, the standard of reference), and two per-volume methods using the average dead-time correction factor of the acquisition applied to all projections before reconstruction (DTCM2) or to the SPECT image after reconstruction (DTCM3). Relative differences in quantification were assessed for various volumes of interest (VOIs) on the phantom and patient SPECT images. In patients, the resulting dosimetry estimates for tissues of interest were also compared between DTCMs.

**Results:**

Both per-volume DTCMs (DTCM2 and DTCM3) were found to be equivalent, with VOI count differences not exceeding 0.8%. When comparing the per-volume post-reconstruction DTCM3 versus the per-projection pre-reconstruction DTCM1, differences in VOI counts and absorbed dose estimates did not exceed 2%, with very few exceptions. The largest absorbed dose deviation was observed for a kidney at 3.5%.

**Conclusion:**

While per-projection dead-time correction appears ideal for QSPECT, post-reconstruction correction is an acceptable alternative that is more practical to implement in the clinics, and that results in minimal deviations in quantitative accuracy and dosimetry estimates, as compared to the per-projection correction.

**Supplementary Information:**

The online version contains supplementary material available at 10.1186/s40658-022-00484-w.

## Introduction

Quantitative single-photon emission computed tomography (QSPECT) is increasingly used for dosimetry in radiopharmaceutical therapy, for which one of the main radionuclides is currently ^177^Lu [1]. We and others have shown that QSPECT dosimetry-based personalisation of ^177^Lu radiopharmaceutical therapy allows to safely escalate activity per cycle and/or cumulative activity during the induction course of peptide receptor radionuclide therapy (PRRT) in patients suffering from neuroendocrine tumours, with encouraging preliminary efficacy results [2, 3]. We previously showed that correcting QSPECT for dead time leads to more accurate quantification and dosimetry and becomes critical when high activity is administered and/or when high retention thereof occurs [4].

Our current dead-time correction method is based on the average observed wide-spectrum count rate during the SPECT acquisition, from which an average dead-time correction factor (DTCF) is deduced and applied to all voxels on the 3D reconstructed volume [5, 6]. However, the observed count rate varies from projection to projection during the acquisition depending on the activity biodistribution and the patient’s morphology. It has been suggested that per-projection dead-time correction may improve accuracy over per-volume correction [7]. However, per-projection correction requires image manipulation before reconstruction, or the use of customised reconstruction software, which makes this approach less practical and potentially less prone to wider adoption and implementation in clinical practice.

This study aimed to compare per-projection versus per-volume dead-time correction methods for QSPECT both in a ^177^Lu-filled phantom and in patients undergoing personalised PRRT, as well as the impact of these methods on absorbed dose estimates for tissues of interest in patients.

## Materials and methods

### SPECT/CT systems

Two dual-head SPECT/CT systems, namely a Symbia T6 (referred to as Symbia) and a Symbia Intevo 6 (referred to as Intevo; Siemens Healthineers, Germany), both equipped with 9.5-mm-thick NaI(Tl) crystals and medium-energy low-penetration collimators were used.

In addition to the ^177^Lu photopeak (208 keV, 20%), lower and upper scatter windows (10% each), three additional energy windows were added to monitor the wide-spectrum count rate (18–680 keV), as previously described [8].

### Phantom and patient acquisitions

A NEMA 2012/IEC 2008 phantom (Biodex Medical Systems, USA) was customised with a similar geometry as the one described in [8]. A large saline bag (500 mL, 50 × background activity concentration) was placed right anteriorly to simulate a large liver lesion and a smaller one (250 mL, 10 × background activity concentration) left posteriorly to simulate a kidney (Table 1). Twenty acquisitions were performed with an initial total ^177^Lu activity of 20.75 GBq on the Intevo system. Phantom acquisitions were performed consecutively with only detector 1 activated and with both detectors activated [8]. All acquisitions were performed with a total number of 96 projections (10 s per projection for the first 13 acquisitions and then 20 s per projection for the seven remaining; 128 × 128 matrix; 4.8 mm pixel). SPECT acquisitions were followed by low-dose CT acquisitions (110 kVp, 70 mAs).

**Table 1:** NEMA phantom initial ^177^Lu activity distribution

Compartment	Volume (ml)	Initial activity (GBq)	Activity concentration (MBq/ml)
Large saline bag	500	13.8	27.6
Small saline bag	250	1.29	5.17
Large sphere	26.5	0.73	27.7
Small sphere	11.5	0.31	26.8
Cylinder	355	0.00	0.00
Remainder of D-shaped compartment	8557	4.63	0.54
Total	9700	20.75	2.14

Data of 14 patients enrolled in our prospective clinical trial of personalised PRRT (NCT02754297) were selected to gather a large range of average observed wide-spectrum count rate (Table 2) [2]. Day-1 QSPECT and Day-3 QSPECT were acquired at 23.3 ± 1.2 and 70.3 ± 0.7 h, respectively, following ^177^Lu-octreotate injection on the Symbia system. Acquisitions were performed with both detectors activated, as described above. The time per projection on Day 1 was either 15 or 20 s, while it was systematically 20 s per projection on Day 3.

**Table 2:** Patients quantitative SPECT data

Patient	Injected activity (GBq)	Time per projection on Day-1 (s)^a^	Estimated activity in FOV Day-1 (GBq)	Averaged *R*_Wo_ Day-1 (cps)	Averaged DTCF Day-1	Estimated activity in FOV Day-3 (GBq)	Averaged *R*_Wo_ Day-3 (cps)	Averaged DTCF Day-3
1	5.92	20	0.43	14,026	1.0077	0.25	7927	1.0044
2	8.10	15	1.82	55,938	1.0324	1.19	37,063	1.0211
3	7.81	15	2.31	62,364	1.0364	1.55	41,518	1.0234
4	17.76	15	3.73	104,385	1.0630	2.48	65,789	1.0381
5	23.46	15	7.38	200,111	1.1330	3.82	113,748	1.0694
6	8.84	15	6.22	169,030	1.1083	4.22	117,650	1.0718
7	28.05	15	9.81	212,717	1.1430	5.22	118,999	1.0729
8	24.83	15	7.09	174,099	1.1126	4.94	121,948	1.0747
9	22.16	20	10.69	211,140	1.1417	6.49	125,913	1.0777
10	21.83	15	11.02	257,596	1.1826	6.19	161,778	1.1029
11	14.65	15	9.24	225,779	1.1544	6.36	167,101	1.1071
12	22.13	15	11.99	293,284	1.2170	8.32	221,955	1.1506
13	28.86	15	16.85^b^	363,755^b^	1.2957^b^	12.22	278,233	1.2017
14	25.53	20	16.12^b^	364,852^b^	1.2971^b^	12.11	297,712	1.2216

*DTCF* dead-time correction factor, *FOV* field of view. *R*_Wo_, observed wide-spectrum count rate

^a^Time per projection on Day-3 was always 20 s

^b^Acquisitions were saturated. Quantification and DTCFs were thus underestimated

The observed count rate was evaluated per projection and per acquisition to exclude saturated (i.e. non quantifiable) phantom data from further studies. An acquisition was considered as saturated if some plateau or abrupt normal variations were observed on the count rate versus projection graph [8].

### Data reconstruction

Projections were reconstructed using SPECTRA Quant (MIM Software Inc., USA) with ordered subset expectation maximisation (four iterations, eight subsets, no post-reconstruction filtering, 128 × 128 matrix, 4.8 × 4.8 × 4.8 mm voxel size), CT-based attenuation correction, triple-energy window scatter correction and resolution recovery.

### Volumes of interest

For the phantom, CT-based volumes of interest (VOIs) were manually drawn around the saline bags and the external contour of the phantom. VOIs of the saline bags were automatically expanded by 0.5 cm in each direction to include spilled-out counts. The background activity within the extended VOI was removed as previously described to obtain the saline bags’ total recovered activity for subsequent analysis [8, 9]. The whole-phantom VOI was automatically expanded by 1 cm [10]. Additionally, a 200-mL background VOI was defined in the D-shaped compartment, far from the spill-out of the other compartments.

For the patients, as detailed in [6], 2-cm VOIs were manually placed in the kidneys and in up to five different dominant tumours (lesions greater than 2 cm in size, with the most prominent uptake). The bone marrow VOI was semi-automatically defined using the CT image: all voxels with Hounsfield units greater than 100 HU corresponding to *L*1–*L*4 vertebras were included.

The mean counts in these VOIs were converted to activity concentration using the calibration factor and the DTCF [10]. For dosimetry, a mono-exponential curve fit was applied to the data of each VOI in addition to the averaged values for the kidneys. This allowed to determine the area under the time–activity concentration curve and to deduce the self-absorbed dose for each tissue using the activity concentration dose factor [2].

### SPECT calibration

The calibration factor (*CF*) and dead-time constant (*τ*) of the Symbia have previously been determined by Frezza et al. [10]. These parameters were determined for the Intevo using the full range of quantifiable phantom data obtained. In brief, the observed wide-spectrum count rate (*R*_Wo_) is expressed in relationship with the activity (*A*) times *R*_Wo_ divided by the observed primary count rate (*R*_Po_) within the phantom VOI [10]. Data points were then fitted to the following equation derived from the Sorenson’s paralysable model to resolve the calibration factor and dead-time constant using Python 3.6 (Lmfit package, least-square minimization) [8, 10, 11]:

$$R_{{{\text{Wo}}}} = {\text{CF}} \cdot A \cdot \frac{{R_{{{\text{Wo}}}} }}{{R_{{{\text{Po}}}} }} \cdot e^{{ - {\text{CF}} \cdot A \cdot \frac{{R_{{{\text{Wo}}}} }}{{R_{{{\text{Po}}}} }} \cdot \tau }}.$$

Once the dead-time constant is deduced, the DTCF can be determined as the ratio of the expected (*R*_We_) on the observed wide-spectrum count rate, using the original Sorenson’s equation:

$$R_{{{\text{Wo}}}} = R_{{{\text{We}}}} \cdot e^{{ - R_{{{\text{We}}}} \cdot \tau }}$$

$${\text{DTCF}} = \frac{{R_{{{\text{We}}}} }}{{R_{{{\text{Wo}}}} }}.$$

Finally, a look-up table is created, with DTCFs corresponding to ascending *R*_Wo_ values.

### Dead-time correction methods

Based on the dead-time constant, a look-up table returning a DTCF value for a given observed wide-spectrum count rate was created. Three dead-time correction methods (DTCM) were tested in the phantom and in patients:

- *DTCM1—pre-reconstruction, per-projection correction* A DTCF was determined for each acquired projection based on the observed wide-spectrum count rate of that projection. Then, the counts of each pixel of the projection’s photopeak, upper, and lower scatter windows were multiplied by that per-projection DTCF before reconstruction. As most SPECT reconstruction software is designed to process only integer counts, it was necessary to round the multiplied counts. Rounding to the closest integer results in inaccurate total number of lost counts injected in the image (e.g. for a DTCF of 1.15, numerous low-activity pixels containing only 1, 2 or 3 counts would be corrected to 1.15, 2.30 and 3.45, respectively, and then systematically rounded down, creating a corrected counts deficit at the image level). Instead, we rounded each pixel counts to the upper integer with a probability equal to its four-digit decimal (using a Python 3.6 script). As this method is not completely deterministic, we performed this process in triplicate, followed by the reconstruction, to evaluate the effect of its randomness component. DTCM1 was considered the reference method, being in principle the most accurate.
- *DTCM2—pre-reconstruction, per-volume correction* Same method as DTCM1, but with all projections corrected using a single DTCF derived from the average observed wide-spectrum count rate of the entire acquisition. The purpose of DTCM2 was to rule out any bias introduced by performing the per-volume correction before versus after reconstruction, as in DTCM3.
- *DTCM3—post-reconstruction, per-volume correction* As detailed in [4–6] and as currently used in our clinics, the DTCF that is derived from the average observed wide-spectrum count rate of the entire acquisition is applied to voxel counts after reconstruction. In this case, there is no need to round to integers, as a float number can be applied subsequently to VOI count data, or conveniently inserted as the “Rescale Slope” parameter (i.e. DTCF multiplied by calibration factor) of the DICOM header of the reconstructed volume converted to the PT modality, enabling to display Bq/mL or standardised uptake values directly in the image viewer.

### Analyses and statistics

For each VOI on the phantom and patient images, the dead-time corrected counts per second according to each DTCM were quantified with the previously determined calibration factor. For the phantom, the estimated quantified activity concentrations for each VOI were also compared with the true activity concentrations. For DTCM1 and DTCM2, the coefficient of variation among the three repetitions was computed for each VOI. The relative differences in counts (and thus in activity concentration) and absorbed doses (in patients) were compared between the DTCMs. All statistics and graphs were generated on R 4.1 (RStudio Inc., Boston, MA, USA, ggplot2 package).

## Results

### Acquisition count rates and data selection

**Fig. 1:**
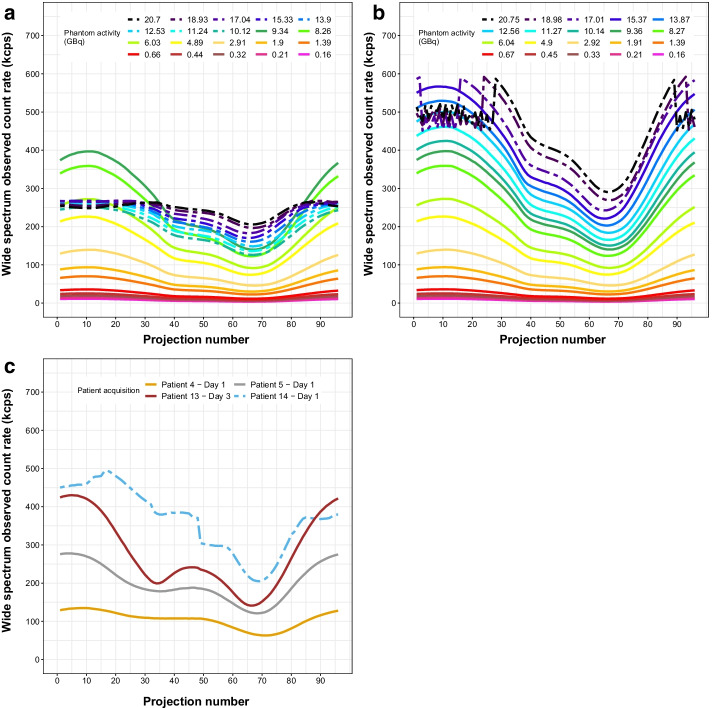
Observed wide-spectrum count rate versus projection number for the phantom (Intevo) with both detectors activated (**a**), only detector 1 activated (**b**), and selected patient acquisitions (**c**, Symbia, both detectors activated). Dashed lines correspond to acquisitions with saturated projections

**Fig. 2:**
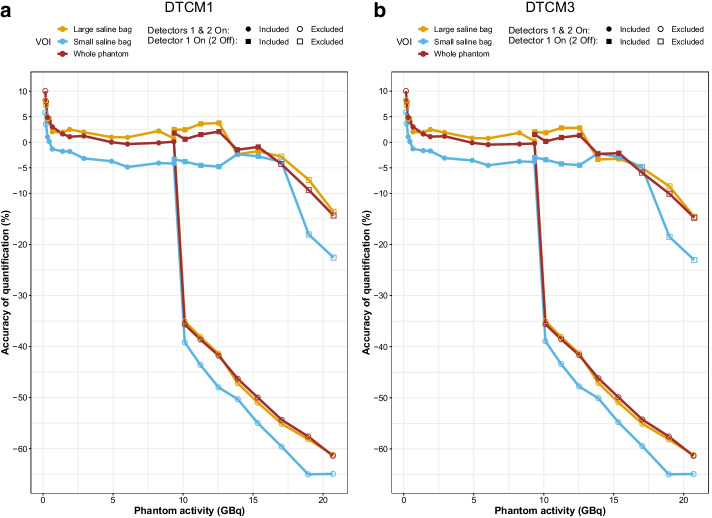
Phantom SPECT quantification errors with both detectors activated (circular points) and only detector 1 activated (detector 2 off, square points) following dead-time correction with DTCM1 (**a**) and DTCM3 (**b**). Hollow data points belong to excluded datasets for which quantification inaccuracies of more than 5% were observed, because of either very low activity or detector saturation

We first examined the observed wide-spectrum count rate per projection for the phantom (20 acquisitions, Fig. 1a, b) and patients (four representative acquisitions, Fig. 1c). In dual-detector mode, the Intevo used for phantom studies saturated at 268 kcps per detector when both detectors were simultaneously exposed to the highest activities (Fig. 1a). Because of the asymmetrical activity distribution in the phantom, it was still possible to obtain non-saturated acquisition data up to 9.34 GBq (i.e. up to 400 kcps on detector 1). When acquiring in single-detector mode (i.e. using only detector 1 over 360°), the absolute saturation level increased to 592 kcps, allowing to quantify up to 15.34 GBq (Fig. 1b). We excluded the two phantom datasets with the lowest activity (0.16 and 0.21 GBq), for which the quantification inaccuracy exceeded 5% in whole phantom or hot objects (Fig. 2). We thus included ten dual-detector acquisitions from 0.32 to 9.34 GBq, plus six single-detector acquisitions from 9.36 to 15.33 GBq for further analysis (*n* = 16).

For the Symbia on which the patient acquisitions were performed, the differential per-detector absolute saturation threshold that we previously observed [8, 10] clearly appeared in two patients on Day 1 after treatment (patients 13 and 14, the latter case illustrated in Fig. 1c). These two acquisitions were excluded from further analysis, resulting in 26 quantifiable acquisitions and 12 valid two-timepoint patients dosimetry studies.

### Calibration factors and dead-time constant

The calibration factor and dead-time constant calculated using the valid reconstructed SPECT phantom data (*n* = 16) and previously described methods [10] were 10.05 ± 0.04 cps/MBq and 0.56 ± 0.14 µs, respectively, for the Intevo. For the Symbia, we used those previously determined by Frezza et al. [10]: 9.36 ± 0.01 cps/MBq and 0.550 ± 0.003 μs, respectively. Considering the close similarity of the dead-time constants and the larger uncertainty of that of the Intevo, we elected to use 0.55 μs across the two systems to generate a common DTCF look-up table for both. The list of phantom acquisitions’ averaged DTCFs is available in Additional file 1: Table S1.

### Validation of pre-reconstruction dead-time correction

To assess the impact of the random component involved in the methods by which the lost counts were injected into projections, DTCM1 and DTCM2 processing and subsequent reconstructions were done in triplicate for each dataset. For all VOIs under study, both in phantom and in patients, and for both DTCM1 and DTCM2, the median and the maximum coefficients of variation of the VOI counts were 0.27% and 1.50%, respectively. This maximum coefficient of variation was found for tumours in patients (*n* = 118), whereas those for all other VOIs did not exceed 0.81%. On this basis, we considered that the randomness with which fractional lost counts are distributed in projections before reconstruction has a negligible impact on quantification.

In principle, when using the average DTCF of the acquisition (i.e. based on the average wide-spectrum count rate from all projections), adding lost counts before (i.e. DTCM2) or after (i.e. DTCM3) the reconstruction should be equivalent. For all VOIs under study, both in the phantom and in patients, the median and maximum relative differences in VOI counts between DTCM2 and DTCM3 were 0.06% and − 0.84%, respectively. We thus concluded that DTCM2 is indeed equivalent to DTCM3, and since it offers no advantage over the latter, it was not studied further.

### Per-volume versus per-projection dead-time correction

**Fig. 3:**
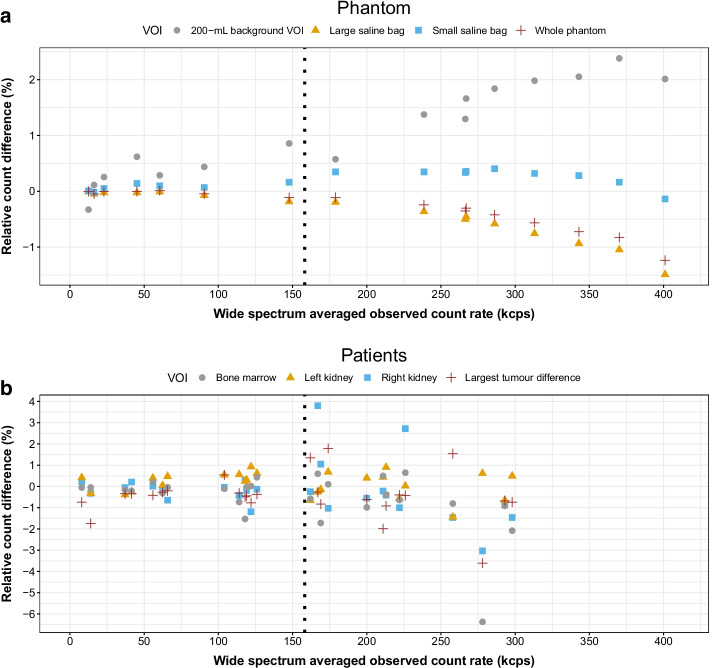
Relative count difference per VOI between SPECT corrected for dead time using DTCM1 versus DTCM3, in function of the observed wide-spectrum acquisition count rate (phantom, **a**; patients, **b**). The dotted line at 158 kcps corresponds to a DTCF equal to 1.1. Tumours are represented by the one tumour per acquisition having the largest difference between DTCM3 versus DTCM1

**Fig. 4:**
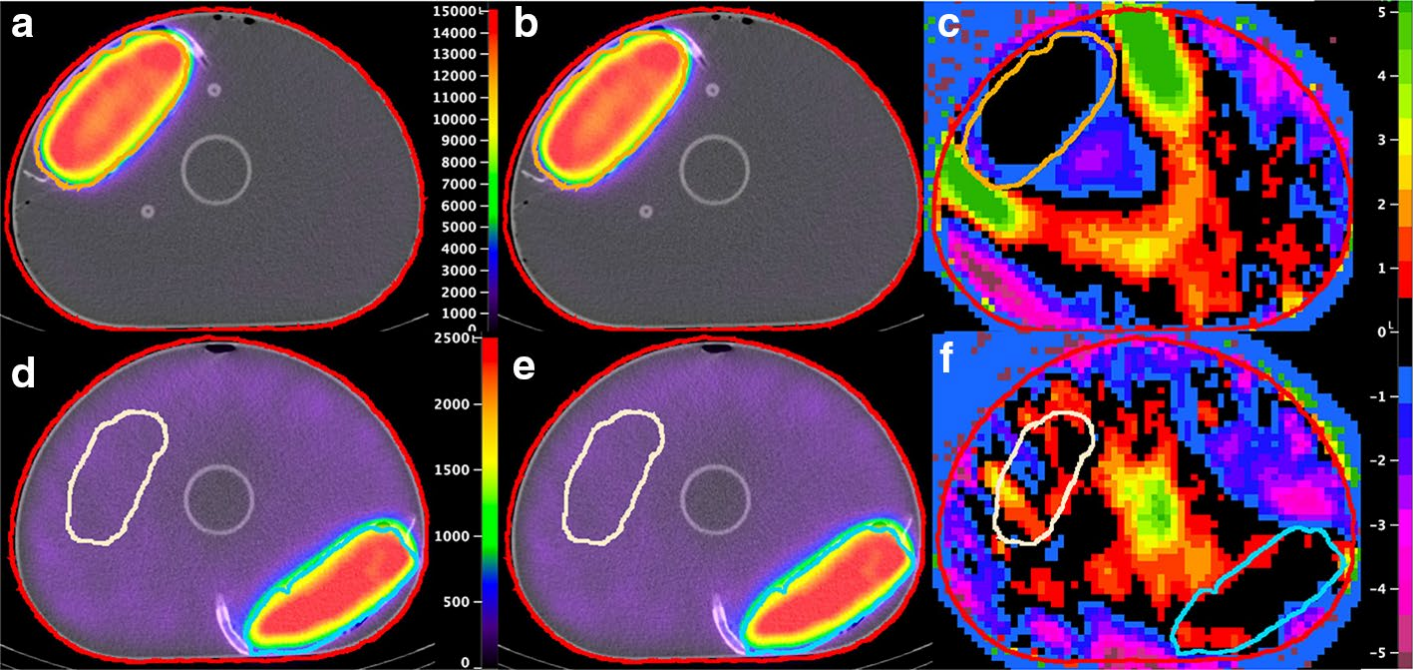
Selected transaxial fusion slices of the phantom (9.34 GBq, average DTCF = 1.1903) corrected with DTCM1 (**a** and **d**) and DTCM3 (**b** and **e**, colour scale in counts), and parametric images of their relative count differences (**c** and **f**, colour scale in per cent). VOIs: whole phantom (red), large saline bag (**a**, **b** and **c**, orange), small saline bag (**d**, **e** and **f**, cyan) background (**d**, **e** and **f**, white). Of note, the peripheral hot objects mimicking target tissues exhibited minimal count differences (mostly black voxels, right)

For the phantom, on a per-VOI basis, the largest median and maximum relative count differences between DTCM3 and DTCM1 were found for the 200-mL background VOI, at 1.08% and 2.38%, respectively. These values were smaller for the other VOIs: − 0.28% and − 1.49% for the large saline bag, 0.16% and 0.40% for the small saline bag, and − 0.18% and − 1.24% for the whole phantom (Fig. 3a). There was a trend towards increasing differences as the count rate increased for most VOIs. Two selected slices of the phantom corrected with DTCM1 and DTCM3 are represented along with parametric images of the per-voxel relative count difference (Fig. 4). Although the voxel-to-voxel differences appear heterogeneously distributed, they are of relatively small amplitude.

In patients, on a per-VOI basis, the largest count difference between DTCM3 versus DTCM1 did not exceed 4% for any VOIs, except for the bone marrow, a VOI with low signal, for which the difference reached − 6.37% in only one case (Fig. 3b). The few occurrences of differences larger than 2% were found at higher counting rates when the DTCF was superior to 1.1.

### Impact of dead-time correction methods on dosimetry

**Fig. 5:**
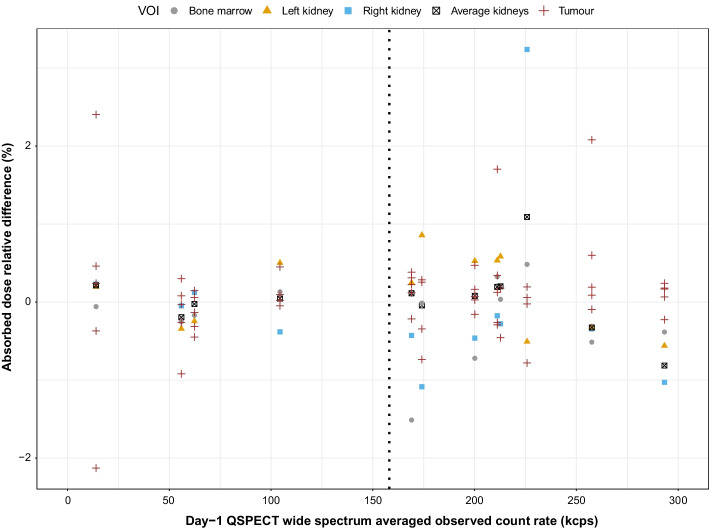
Relative difference between self-absorbed dose estimates computed from two-timepoint QSPECT corrected for dead time using DTCM3 versus DTCM1, in function of the Day-1 observed wide-spectrum count rate. The dotted line at 158 kcps corresponds to a DTCF equal to 1.1

In 12 patients with a valid two-timepoint SPECT/CT study, the median (min–max) self-absorbed doses were 1.29 (0.23–3.62) Gy for the bone marrow, 6.11 (4.01–9.79) Gy for the left kidney, 5.18 Gy (2.36–7.04) for the right kidney, 5.98 Gy (3.19–7.80) for both kidneys averaged, and 39.76 Gy (1.13–155.68, *n* = 54) for tumours. The largest median and maximum relative dose differences between DTCM1 versus DTCM3 were 0.31% and 3.24%, respectively (Fig. 5). Of 102 datapoints, only nine exceeded ± 1%. These were more frequent when DTCF was superior to 1.1.

## Discussion

We have previously shown that dead-time correction is essential for accurate QSPECT in the context of dosimetry-based personalised radiopharmaceutical therapy [4]. Few dead-time correction methods have been proposed for QSPECT [5, 7, 10, 12–14]. Most of them use a single DTCF that is applied to the reconstructed image. Here, we observed very small relative differences between per-projection (DTCM1) and per-volume (DTCM3) dead-time correction methods, in terms of both SPECT quantification and absorbed dose estimates during ^177^Lu radiopharmaceutical therapy. These differences were vastly within 1% and at most a few per cent.

To our knowledge, only Cohalan et al*.* have previously performed a comparative study of per-projection versus per-volume dead-time correction based on two SPECT acquisitions of an asymmetrical 2.3 GBq ^99m^Tc-filled phantom causing ~ 20% dead time (i.e. DTCF≈1.25) [7]. They reported an average quantification difference of 1.7% between methods among few VOIs. Extrapolating from a figure in their paper, the corresponding median and maximum differences were approximately 1% and 3%, respectively. Their results thus appear consistent with ours, especially in cases with a DTCF larger than 1.1 (right of the dashed line, Fig. 3a, b). Interestingly, the authors used a Discovery NM/CT 670 SPECT/CT system (GE Healthcare), which has a larger dead-time constant than the system considered in this study (1.74 vs. 0.55 μs, respectively) [8]. Such a system may potentially increase the amplitude of dead-time variation between projections for objects or patients with a markedly asymmetrical activity distribution. Nevertheless, the differences between per-volume and per-projection corrections remained small, and within a similar range than those we observed. Some strengths of our study are that we used ^177^Lu instead of ^99m^Tc and that we also analysed the impact of dead-time correction methods on patient QSPECT data and dosimetry estimates.

We used a different approach than that of Cohalan et al*.* to correct projections for dead time. To achieve this, they inserted a script into the reconstruction loop of commercial software (HybridRecon, Hermes Medical Solutions, Sweden), while we injected lost counts into the acquisition file prior to reconstruction. However, this required in-house scripting in both cases and implied having access to editable reconstruction software or adding an image manipulation step, respectively. Conversely, post-reconstruction dead-time correction is more convenient to perform. While a script or batch file may ease computation of the average wide-spectrum count rate and the automatic look-up of the DTCF, these tasks can also easily be accomplished using common nuclear medicine and spreadsheet software.

We concur that, ideally, the most physically accurate dead-time correction method should be used, i.e. per-projection. Eventually, the latter may become standard in commercial QSPECT/CT systems that are being developed. Until then, post-reconstruction per-volume correction appears an appropriate and practical alternative that may be more easily amenable to wider implementation in the clinics. This is applicable to NaI crystal-based SPECT/CT systems. Of note, as newly introduced CZT-based SPECT/CT systems may exhibit less dead time, they should be even less influenced by the choice of the DTCM [15].

## Conclusion

As compared to per-projection dead-time correction, per-volume dead-time correction using a DTCF based on the average acquisition wide-spectrum count rate results in small differences in SPECT quantification and dosimetry estimates that rarely exceed 2%. Post-reconstruction dead-time correction, which is easier to implement, thus appears adequate for use in personalised radiopharmaceutical therapy.

## Supplementary Information

**Additional file 1.** NEMA phantom acquisitions total activity and averaged DTCF.

## Data Availability

Please contact the corresponding author for the data used in this manuscript.

## Abbreviations

CF: Calibration factor
*τ*: Dead-time constant
DICOM: Digital imaging and communications in medicine
DTCF: Dead-time correction factor
DTCM: Dead-time correction method
FOV: Field of view
PRRT: Peptide receptor radionuclide therapy
QSPECT: Quantitative single-photon emission computed tomography
*R*_We_: Expected wide-spectrum count rate
*R*_Wo_: Observed wide-spectrum count rate
VOI: Volume of interest
