# Supplementary material for: Impact of the dead-time correction method on quantitative 177Lu-SPECT (QSPECT) and dosimetry during radiopharmaceutical therapy
Source: EJNMMI Phys. 2022 Aug 17;9:54. doi: 10.1186/s40658-022-00484-w (PMC9385894; doi:10.1186/s40658-022-00484-w)
Supplement: Supplementary file 1 — Additional file 1. NEMA phantom acquisitions total activity and averaged DTCF. [file 40658_2022_484_MOESM1_ESM.docx]

**Supplemental Table 1** NEMA phantom acquisitions total activity and averaged DTCF

| Acquisition | Total Activity (GBq) | Detectors 1 and 2 On  Averaged DTCF | Detector 1 On (2 Off)  Averaged DTCF |
| --- | --- | --- | --- |
| 01 | 20.70 | 1.1708 ^a^ | 1.4075 ^a^ |
| 02 | 18.93 | 1.1689 ^a^ | 1.3868 ^a^ |
| 03 | 17.04 | 1.1657 ^a^ | 1.3656 ^a^ |
| 04 | 15.33 | 1.1593 ^a^ | 1.3447 |
| 05 | 13.90 | 1.1536 ^a^ | 1.3040 |
| 06 | 12.53 | 1.1460 ^a^ | 1.2707 |
| 07 | 11.24 | 1.1372 ^a^ | 1.2370 |
| 08 | 10.12 | 1.1291 ^a^ | 1.2094 |
| 09 | 9.34 | 1.1903 | 1.1909 |
| 10 | 8.26 | 1.1651 | 1.1657 |
| 11 | 6.03 | 1.1162 | 1.1168 |
| 12 | 4.89 | 1.0925 | 1.0931 |
| 13 | 2.91 | 1.0536 | 1.0542 |
| 14 | 1.90 | 1.0352 | 1.0352 |
| 15 | 1.39 | 1.0256 | 1.0262 |
| 16 | 0.66 | 1.0127 | 1.0127 |
| 17 | 0.44 | 1.0088 | 1.0088 |
| 18 | 0.32 | 1.0072 | 1.0072 |
| 19 | 0.21 | 1.0050 | 1.0050 |
| 20 | 0.16 | 1.0039 | 1.0039 |

^a^ Acquisitions were saturated. Quantification and DTCFs were thus underestimated.

DTCF = dead-time correction factor
